# A Common Human Brain-Derived Neurotrophic Factor Polymorphism Leads to Prolonged Depression of Excitatory Synaptic Transmission by Isoflurane in Hippocampal Cultures

**DOI:** 10.3389/fnmol.2022.927149

**Published:** 2022-06-23

**Authors:** Riley A. Williams, Kenneth W. Johnson, Francis S. Lee, Hugh C. Hemmings, Jimcy Platholi

**Affiliations:** ^1^Department of Anesthesiology, Weill Cornell Medicine, New York, NY, United States; ^2^Department of Pharmacology, Weill Cornell Medicine, New York, NY, United States; ^3^Department of Psychiatry, Sackler Institute for Developmental Psychobiology, Weill Cornell Medicine, New York, NY, United States; ^4^Feil Family Brain and Mind Research Institute, Weill Cornell Medicine, New York, NY, United States

**Keywords:** BDNF, isoflurane, single nucleotide polymorphism, synaptic vesicle exocytosis, synaptic plasticity

## Abstract

Multiple presynaptic and postsynaptic targets have been identified for the reversible neurophysiological effects of general anesthetics on synaptic transmission and neuronal excitability. However, the synaptic mechanisms involved in persistent depression of synaptic transmission resulting in more prolonged neurological dysfunction following anesthesia are less clear. Here, we show that brain-derived neurotrophic factor (BDNF), a growth factor implicated in synaptic plasticity and dysfunction, enhances glutamate synaptic vesicle exocytosis, and that attenuation of vesicular BDNF release by isoflurane contributes to transient depression of excitatory synaptic transmission in mice. This reduction in synaptic vesicle exocytosis by isoflurane was acutely irreversible in neurons that release less endogenous BDNF due to a polymorphism (BDNF Val66Met; rs6265) compared to neurons from wild-type mice. These effects were prevented by exogenous application of BDNF. Our findings identify a role for a common human BDNF single nucleotide polymorphism in persistent changes of synaptic function following isoflurane exposure. These short-term persistent alterations in excitatory synaptic transmission indicate a role for human genetic variation in anesthetic effects on synaptic plasticity and neurocognitive function.

## Introduction

General anesthetics are distinguished by route of administration as inhalational or volatile anesthetics (VA) and intravenous (IV) anesthetics, and by their diverse chemical structures, molecular targets, and binding sites. Although mechanistically distinct, all general anesthetics alter synaptic transmission by acting both presynaptically and postsynaptically (MacIver, 2014; Hemmings et al., 2019). While these effects are anticipated to be fully reversible upon drug elimination, concerns that exposure to general anesthesia in vulnerable populations can induce lasting impairments in synaptic plasticity and cognitive function remain. Perioperative neurocognitive disorder (PND; Evered et al., 2018) affects up to 41% of patients older than 60 years (Monk et al., 2008; Berger et al., 2015; Terrando et al., 2015), and additional risk factors include pre-existing cognitive impairment or disease (Feinkohl et al., 2017) as well as education level and socioeconomic status (Silbert et al., 2015). Several reports show a possible association between a specific genotype and PND; polymorphisms of the human gene C-reactive protein (Zhang et al., 2015), P-selectin (Mathew et al., 2007), and platelet glycoprotein IIIa (Mathew et al., 2001) suggest an additional vulnerability in genotype may increase susceptibility to PND. Thus, identifying and addressing the role of genetic variation in preoperative risk assessment and recovery from anesthesia will facilitate individualized mechanism-based use of specific anesthetic agents based on patient factors (precision anesthesia).

Brain-derived neurotrophic factor (BDNF) is a neuromodulator synthesized as a precursor (proBDNF) that is cleaved to its mature form (mBDNF) and released to mediate divergent actions on neuronal survival, neuron structure, and synaptic plasticity (Teng et al., 2005; Cowansage et al., 2010). In the adult brain, mBDNF regulates the density and morphology of dendritic spines (Poo, 2001; Chapleau et al., 2009), promotes neuronal survival and enhances synaptic plasticity (Soppet et al., 1991; Mizuno et al., 2003; Yang et al., 2016). Studies support a role for down-regulation of mBDNF by anesthetics; in humans, both intravenous (propofol) and volatile (isoflurane) anesthetics reduce plasma BDNF concentration intraoperatively and 24 h after surgery (Vutskits et al., 2008), while epigenetic enhancement of BDNF signaling improves cognitive impairment induced by isoflurane anesthesia in aged rats (Ji et al., 2014).

Human carriers of the common BDNF Val66Met single nucleotide polymorphism (SNP; rs62645; 30% prevalence; Petryshen et al., 2010) have reduced hippocampal volume, altered synaptic plasticity, and impaired hippocampus-dependent learning and memory (Egan et al., 2003). This SNP results in abnormal intracellular trafficking of BDNF leading to lower regulated secretion of mBDNF (Hariri et al., 2003; Szeszko et al., 2005; Bath and Lee, 2006). This allele is also associated with reduced preoperative cognitive function leading to attenuated cortical activity during surgery under volatile anesthesia suggestive of further cognitive decline and susceptibility to PND (Mulholland et al., 2014; Silbert et al., 2015; Giattino et al., 2017). Understanding the pharmacogenetic interaction between this common SNP and general anesthesia should provide novel insights into the neurocognitive effects of anesthetics, but the cellular and molecular mechanisms behind the contribution of BDNF and the Val66Met polymorphism to synaptic deficits following general anesthesia have not been explored.

Mechanisms for acute depression of excitatory synaptic transmission by general anesthesia include reduction of neuronal excitability (Pittson et al., 2004), action potential conduction (Berg-Johnsen and Langmoen, 1986; Mikulec et al., 1998; Wu et al., 2004; Ouyang and Hemmings, 2005), inhibition of Ca^2+^ influx (Baumgart et al., 2015; Koyanagi et al., 2019) and synaptic vesicle (SV) exocytosis (Westphalen and Hemmings, 2003, Westphalen and Hemmings, 2006; Torturo et al., 2019), and/or blockade of postsynaptic glutamate receptors (Dickinson et al., 2007). Accordingly, we have shown differential reduction of evoked release of various major CNS neurotransmitters by volatile anesthetics (Miao et al., 1995; Schlame and Hemmings, 1995; Hemmings et al., 2005). For example, isoflurane more potently inhibits glutamatergic compared to GABAergic or dopaminergic action-potential (AP)-evoked synaptic vesicle exocytosis in dissociated primary neurons (Baumgart et al., 2015; Torturo et al., 2019). These observations are similar to findings in cortical GABAergic neurons *in vivo* (Marvin et al., 2018, 2019).

Neurotransmitter release is tightly coupled to Ca^2+^ entering the presynaptic bouton (Kim and Ryan, 2013). Various anesthetics differentially act on presynaptic Ca^2+^ signaling via ion channels or vesicle fusion mechanisms to inhibit exocytosis (Pocock and Richards, 1992; Schlame and Hemmings, 1995) by attenuating release probability and number of functional release sites (Kitamura et al., 2003; Wang et al., 2020). Candidate targets include Na^+^, Ca^+^, and K^+^ channels (Study, 1994; Franks and Honoré, 2004; Herold and Hemmings, 2012), neurotransmitter transporters (Westphalen and Hemmings, 2003; Cechova and Zuo, 2006) and SNARE exocytotic proteins (Nagele et al., 2005; Herring et al., 2009; Xie et al., 2013). Yet these presynaptic sites of action do not fully explain anesthetic-induced effects on synaptic transmission.

Activity-dependent mBDNF secretion enhances neurotransmitter release by multiple mechanisms that increase intracellular Ca^2+^ (Gärtner et al., 2006), directly act on vesicle machinery (Jovanovic et al., 2000; Thakker-Varia et al., 2001), and/or increase neurotransmitter replenishment and release (Tyler and Pozzo-Miller, 2001; Tyler et al., 2006). Mutation or selective deletion of BDNF results in fewer docked vesicles (Pozzo-Miller et al., 1999), reduced release probability (Lin et al., 2018), and reduced expression of synaptic vesicle proteins, such as synaptobrevin and synaptophysin, that regulate neurotransmitter release (Pozzo-Miller et al., 1999). These observations suggest that regulation of neurotransmitter release by mBDNF may be a novel presynaptic anesthetic mechanism. We tested the hypothesis that BDNF signaling contributes to isoflurane inhibition of excitatory synaptic transmission and that these effects are enhanced in neurons with reduced BDNF secretion.

## Materials and Methods

This study was performed in strict accordance with the recommendations in the Guide for the Care and Use of Laboratory Animals of the National Institutes of Health. The Institutional Animal Care and Use Committee (IACUC) of Weill Cornell Medicine specifically approved this study under protocol #2015-0023. All animals were handled according to this approved protocol and followed ARRIVE guidelines. All surgical procedures were terminal and anesthesia with isoflurane was used to prevent animal suffering.

### Reagents

Isoflurane was obtained from Abbott (Abbott Park, IL) and all other reagents were sourced as indicated. Rat proBDNF-pHluorin was from Ed Chapman (University of Wisconsin, Madison, WI), and rat vGlut1-pHluorin was from Timothy Ryan (Weill Cornell Medicine, New York, NY).

### Hippocampal Neuron Culture and Transfection

Rat and mouse hippocampal cells were cultured according to Calabrese and Halpain (2015). Briefly, whole hippocampi were dissected from embryonic day 16–18 (E16–18). Sprague Dawley rat or mouse embryos, and cells dissociated with papain, cultured on 25 mm glass coverslips (Carolina Biological, Burlington, NC) in 35-mm dishes (Corning, Durham, NC) at a density of 100,000–150,000 cells/mm^2^, and maintained in Neurobasal medium (GIBCO, Grand Island, NY) supplemented with SM1 (Stem Cell Technologies, Vancouver, BC, Canada) and 0.5 mM L-glutamine (Sigma-Aldrich, St. Louis, MO). The BDNF Val66Met mice (Chen et al., 2006) were generated by Francis Lee. For mouse cultures, heterozygous (Val66Met) male and female mice were paired to yield Val66Val (wt), Val66Met, and Met66Met genotypes. Individual mouse pups were genotyped and cultured separately. Cultures were transfected at 7 days *in vitro* (DIV) by calcium phosphate precipitation with 6–10 μg CAG promoter driving mCherry cDNA to allow visualization of neuronal morphology mixed with BDNF-pHluorin or vGlut1-pHluorin cDNA to visualize exocytosis as described (Köhrmann et al., 1999). Cells were incubated with the transfection mixture for 2. 5–3 h in 95% air 5% CO_2_ at 37°C, washed twice with pre-warmed HBS [in mM: 135NaCl, 4KCl, 1Na_2_HPO_2_, 2CaCl_2_, 1MgCl_2_, 10 glucose, and 20 HEPES (pH 7.35)], and replaced with Neurobasal medium. Cells were used for live cell-imaging at 14–16 DIV.

### Drug Treatments

Isoflurane was used at aqueous concentrations equivalent to 0.25–2 times minimum alveolar concentration (MAC) in rat (Taheri et al., 1991) as a clinically relevant dose range (1 MAC is ED_50_). Saturated stock solutions were diluted to working concentrations for superfusion through Teflon tubing using gas-tight glass syringes. Coverslips were placed in a heated (37°C) perfusion chamber (260 μl) and Tyrode’s solution [in mM: 119 NaCl, 2.5 KCl, 2 CaCl_2_, 2 MgCl_2_, 25 HEPES, and 30 glucose (pH 7.4)] ± isoflurane was delivered using a custom pressurized, inline heated, gas-tight superfusion system (Hemmings et al., 2005) at 2 ml/min (equilibration time constant of ∼8 s). Cells were equilibrated with control or anesthetic solutions prior to start of experiments and anesthetic concentrations from bath and syringe samples were confirmed by gas chromatography (Herold et al., 2009). TrkB-Fc (1 μg/ml; ED_50_ = 0.1–0.4 μg/ml; R and D Systems, Minneapolis, MN) and recombinant BDNF (75–100 ng/ml; PeproTech, Rocky Hill, NJ; Yang et al., 2016) were added 5 min prior to stimulation. Drug concentrations were based on cell type, stimulation frequency, and incubation times (Rex et al., 2007).

### Measurement of Exocytosis

**FIGURE 1:**
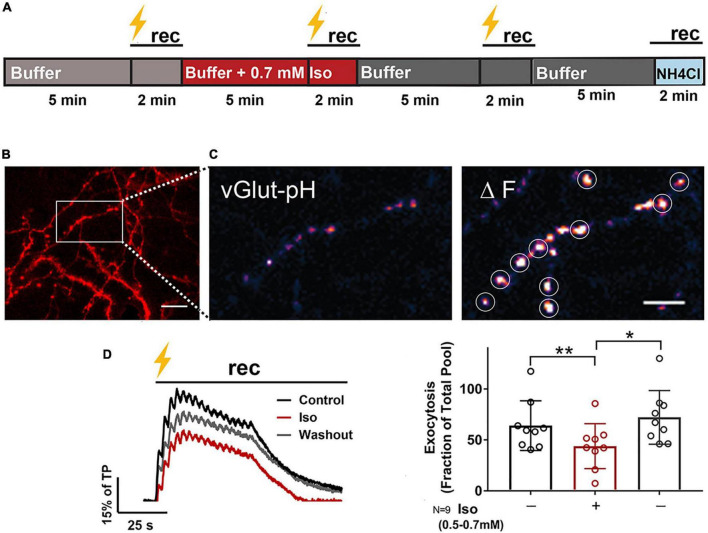
Isoflurane reversibly inhibits electrically-evoked synaptic vesicle exocytosis. Rat hippocampal neuron cultures (16DIV) transfected with vGlut-pH and mCherry were perfused with 0.5–0.7 mM isoflurane or control buffer with tetanic stimulation (lightning bolt) and imaged as indicated in **(A)**. Cells were equilibrated with control buffer or buffer with isoflurane for 5 min prior to stimulation and time-lapse recording for 2 min. Representative images showing dendritic arbor and axons of a control neuron (**B**, mCherry). Inset shows single boutons (vGlut-pH) before (**C**, left) and after (**C**, right) tetanic stimulation with defined ROIs used for analysis (white circles). Representative paired traces of electrically-evoked synaptic vesicle exocytosis (black bar, tetanic stimulation) of ROIs (average of ∼50) from a single neuron under control, isoflurane, and washout conditions during 2 min recording (**D**, left). Peak synaptic vesicle exocytosis was reduced by isoflurane (**D**, right; peak fluorescence) (**p < 0.01 by one-way multiple comparisons ANOVA with Dunnett’s *post-hoc* test) and recovered following washout (**D**, right; peak fluorescence) (*p < 0.05 by one-way multiple comparisons ANOVA with Dunnett’s *post-hoc* test). Data are mean±SD%; *n* = 9 neurons per experimental group and 405–450 boutons. Scale bar = 5 μm.

Live-cell time-lapse fluorescence images at 10 Hz were collected using a 40 × 1.3 (NA) Plan APO oil immersion objective and a Zeiss (Thornwood, NY) Axio Observer 7 microscope equipped with a chamber temperature-controlled at 37°C (Warner Instruments, Hamden, CT). Cells were excited using Colibri 7 solid state LED lamps (469 or 555 nm) and fluorescence emission was selected through the 514/30 or 592/25 nm band-pass filters (Zeiss, Thornwood, NY). A series of time-lapse images was acquired every 50 ms for 2 min with an Andor iXon 888 electron multiplying CCD camera (Andor, South Windsor, CT) using Zen 2.3 imaging software (Zeiss, Thornwood, NY). The time courses of pHluorin fluorescence responses to tetanic stimulation [2 min; 16 bursts of 50 action potentials (AP) at 50 Hz every 2.5 s] were measured for paired control and treatment conditions in Tyrode’s solution in the following order: (1) BDNF-pH: 5 min control solution equilibration; 5 s baseline recording; 2 min depolarizing tetanic stimulation in control solution recording; 5 min isoflurane equilibration; 5 s baseline isoflurane recording; 2 min depolarizing tetanic stimulation in isoflurane solution recording; 5 min washout in control solution; 50 mM NH_4_Cl alkalization; (2) vGlut1-pH: 5 min control solution equilibration; 5 s baseline recording; 2 min depolarizing tetanic stimulation in control solution recording; 5 min isoflurane equilibration; 5 s baseline isoflurane recording; 2 min depolarizing tetanic stimulation in isoflurane solution recording; 5 min washout in control solution; 2 min depolarizing tetanic stimulation in control solution recording; 50 mM NH_4_Cl alkalization (Figure 1A). Electrical stimulation was performed using platinum-iridium electrodes; and multiple successive time-control stimulations for tetanic pulses were used to rule out decay over time (Supplementary Figure 1).

### Image and Statistical Analysis

As secretion of BDNF is asynchronous (Figure 3B), we modified our analysis from Shimojo et al. (2015) to quantify BDNF release as number of events as opposed to change in fluorescence normalized to NH_4_Cl since each event leads to multiple maximum peaks across acquisitions. Regions of interest (ROIs) were identified using a graphical user interface-based software from Matlab (Fluorosnnap; University of Pennsylvania) where fluorescence intensity was calculated for each ROI based on differences in fluorescence from baseline. Each ROI was subjected to a signal-to-noise (SNR) calculation based on its response to the first control electrical stimulation, and ΔF was calculated as the difference in average intensities between F_peak_ and F_baseline_ and counted as an event if mean ΔF was>4 SD above baseline. For vGlut1-pH, transfected boutons were selected as ROIs based on their response to NH_4_Cl and/or labeling with mCherry. Fluorescence data were analyzed in ImageJ with a custom plug-in^1^ and each transfected bouton was subjected to a SNR calculation based on its response to the first control electrical stimulation, and ΔF/F calculated as the difference in average intensities between F_peak_ and F_baseline_/F_baseline_. Boutons with SNR>4 SD were used in the analysis. Baseline measurements were calculated from 50 frames (5 s) preceding each treatment condition prior to stimulation and maximum peak values from 10 ROIs were averaged following evoked release for F_peak_ and quantification and analysis. Release probability was analyzed as mean fusion events (BDNF; Shimojo et al., 2015) or mean (vGlut1; Balaji and Ryan, 2007) ΔF/F normalized to total vesicle pool determined by alkalization with 50 mM NH_4_Cl to compensate for differences in density of available vesicles across independent imaging experiments. Selection of ∼30–50 ROIs from each neuron and experimental condition (control, drug, washout) included both distal and proximal regions relative to the soma for BDNF-pH and axonal boutons for vGlut1-pH. Data are expressed as mean±SD. Drug effects are shown as a percentage of total pool release or control response to allow quantification of inhibition or potentiation. Statistical significance was determined by unpaired two-tailed Student’s *t*-test or one-way ANOVA with Dunnett’s *post-hoc* test, with *p*< 0.05 considered significant. Statistical analysis and graph preparation were performed using GraphPad Prism v7.05 (Graphpad Software, Inc., San Diego, CA).

## Results

### Isoflurane Reversibly Inhibits Excitatory Synaptic Vesicle Exocytosis

Reductions in SV exocytosis by general anesthetics vary with type of neuron (Baumgart et al., 2015; Torturo et al., 2019) and can be mechanistically distinct based on stimulation frequency (Baumgart et al., 2015; Wang et al., 2020). With short depolarizing pulses, anesthetics reduce Ca^2+^ influx independent of Ca^2+^-exocytosis coupling (Baumgart et al., 2015; Wang et al., 2020), while with longer depolarizations anesthetics directly inhibit the SV exocytotic machinery downstream of Ca^2+^ influx (Wang et al., 2020). With short physiological stimulations, isoflurane transiently inhibits transmitter release from hippocampal glutamatergic and GABAergic or ventral tegmental area (VTA) dopaminergic neurons (Baumgart et al., 2015; Torturo et al., 2019). Patterns of both high and low brain activity are observed under anesthesia in both rodents and humans (Brown et al., 2010; Fleischmann et al., 2018), but little research has focused on long, repetitive input, particularly in the hippocampus, an area critical for synaptic plasticity and BDNF signaling.

Rat hippocampal neurons were exposed to isoflurane (0.53–0.7 mM; ∼1.5–2 MAC) and stimulated with high-frequency tetanic pulses (16 bursts of 50 action potentials at 50 Hz every 2.5 s; Kolarow et al., 2007; Matsuda et al., 2009) to examine the effects of isoflurane on vGlut-pH SV exocytosis (Figure 1A and Supplementary Movie 1). Presynaptic boutons were visualized using mCherry (Figure 1B) and exocytosis at glutamatergic nerve terminals was measured by the fluorescence change in pH-sensitive pHluorin fused to the luminal domain of the vesicular glutamate transporter (vGlut-pH; Balaji and Ryan, 2007 and Figure 1C). Fluorescence responses were normalized to total SV pool by NH_4_Cl alkalization and to control for between neuron variability. The vGlut-pH biosensor reliably measured SV exocytosis over time with minimal decay over the course of three control stimulations (Supplementary Figure 1). In glutamatergic neurons, stimulated exocytosis was 64% of total pool (TP), which was reduced to 44% of TP by 0.7 mM isoflurane (31% inhibition), a clinically relevant concentration ∼ 1.4 times the EC_50_ for general anesthesia in rat (Taheri et al., 1991). Exocytosis returned to control levels (72% of TP) within 3 min of isoflurane washout under the same stimulation conditions (Figures 1A,D). This recovery of SV release was stable unless cell health was compromised due to photobleaching or toxicity and not included in the analysis. These results show that compared to lower depolarization frequencies (Baumgart et al., 2015; Torturo et al., 2019), release as a fraction of total pool is greater with high frequency stimulation but is comparably reversible and recovers following isoflurane washout.

### Brain-Derived Neurotrophic Factor Contributes to Excitatory Synaptic Vesicle Exocytosis

**FIGURE 2:**
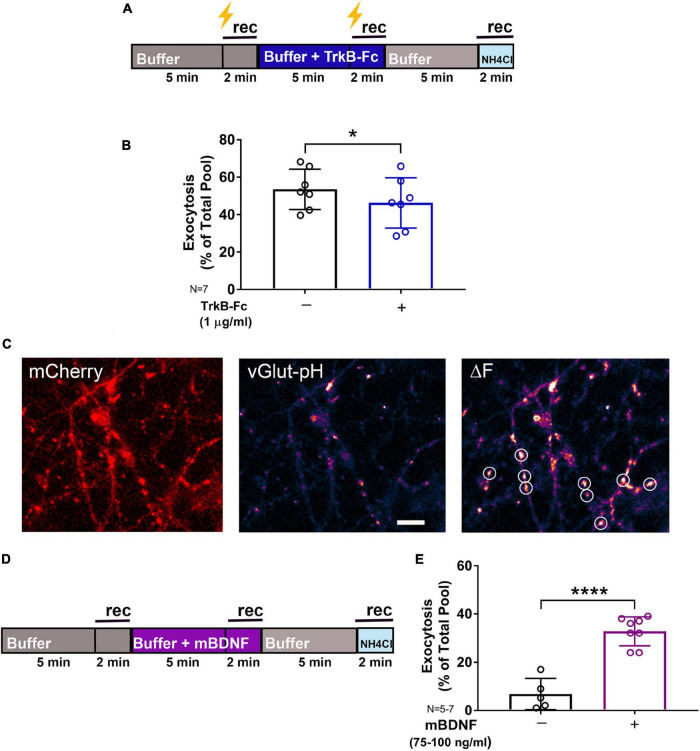
Endogenous BDNF contributes to synaptic vesicle exocytosis. Rat hippocampal neuron cultures (16DIV) transfected with vGlut-pH and mCherry were perfused with TrkB-Fc (1 μg/ml) prior to tetanic stimulation (lightning bolt; **A**) or mBDNF with no stimulation **(D)** and imaged as indicated. Quantification of electrically-evoked synaptic vesicle exocytosis shows that pretreatment with the BDNF binding protein TrkB-Fc (1 μg/ml) for 5 min prior to tetanic stimulation reduced exocytosis **(B)** (*p < 0.05 by Student’s paired *t*-test). Representative images of rat hippocampal neurons (16DIV) transfected with mCherry (**C**, left) and vGlut-pH showing single boutons before (**C**, middle) and after (**C**, right) addition of exogenous mBDNF (75–100 ng/ml) with defined ROIs used for analysis (white circles). Synaptic vesicle exocytosis was increased by mBDNF without stimulation **(E)** (****p < 0.0001 by Student’s paired *t*-test). Data are mean±SD; *n* = 5–7 neurons per experimental group and 250–350 boutons. Scale bar = 5 μm.

In the mature brain, mBDNF is a key modulator of synaptic plasticity that enhances glutamate release by multiple mechanisms: activation of phospholipase Cγ (PLCγ) increases intracellular Ca^2+^ (Gärtner et al., 2006); phosphorylation of synapsin by mitogen-activated protein kinase (MAPK) (Jovanovic et al., 2000) and increased Rab3 expression (Thakker-Varia et al., 2001) enhance exocytosis; and activation of the actin motor complex increases neurotransmitter replenishment and release (Tyler and Pozzo-Miller, 2001; Tyler et al., 2006; Park and Poo, 2013). We tested the contribution of BDNF to SV exocytosis in response to high-frequency tetanic pulses using TrkB-Fc (1 μg/ml), a chimeric protein containing the extracellular domain of TrkB that binds and sequesters endogenous BDNF. Reduction of extracellular BDNF levels by TrkB-Fc inhibited tetanic stimulation-evoked SV exocytosis by 17% as quantified using vGlut-pH in rat hippocampal neurons (Figures 2A,B). Quantification of vGlut-pH without electrical stimulation confirmed that exogenous addition of mBDNF (75–100 ng/ml) significantly increased basal SV exocytosis (Figures 2C–E). *In vivo* studies support a role for down-regulation of mBDNF by anesthetics (Vutskits et al., 2008; Ji et al., 2014), but whether BDNF and anesthetic regulation of synaptic transmission interact has not been tested.

**FIGURE 3:**
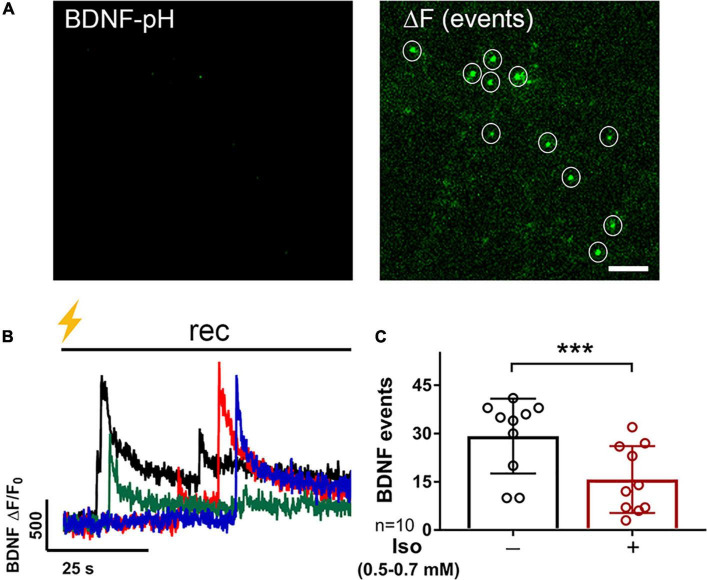
BDNF exocytosis is reduced by isoflurane. Representative images of rat hippocampal neurons (18DIV) transfected with mCherry (not shown) and BDNF-pH showing single BDNF exocytotic events (white circles) before (**A**, left) and after (**A**, right) tetanic stimulation (**B**, lightning bolt) showing asynchronous release of BDNF-pH (**B**, left). Individual traces represent single BDNF exocytosis shown in different colors. Quantification of BDNF exocytosis defined using fluorescence responses to tetanic stimulation showed inhibition by 0.5–0.7 mM isoflurane **(C)** (***p < 0.001 by Student’s paired *t*-test). Data are mean ± SD; *n* = 10 neurons per experimental group and 23–113 BDNF events selected for each condition. Scale bar = 5 μm.

### Attenuation of Brain-Derived Neurotrophic Factor Release by Isoflurane Contributes to Depression of Excitatory Synaptic Vesicle Exocytosis

**FIGURE 4:**
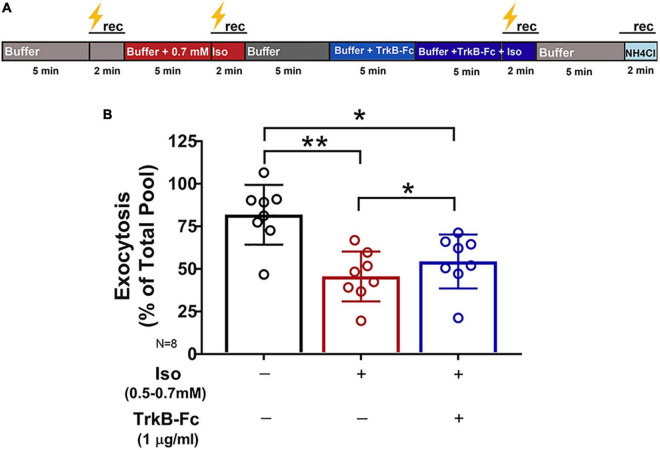
Inhibition of synaptic vesicle exocytosis by isoflurane is attenuated by reducing endogenous BDNF. Rat hippocampal neuron cultures (16DIV) transfected with vGlut-pH and mCherry were perfused with control buffer, 0.5–0.7 mM isoflurane, or TrkB-Fc (1 μg/ml) + 0.5–0.7 mM isoflurane with tetanic stimulation (lightning bolt) as indicated in **(A)**. **(B)** Synaptic vesicle exocytosis evoked by tetanic stimulation was reduced by isoflurane (0.5–0.7 mM) (**p< 0.01 by multiple comparisons one-way ANOVA with Dunnett’s *post-hoc* test). Reduced exocytosis observed with isoflurane exposure was attenuated by pretreatment with TrkB-Fc (1 μg/ml) for 5 min prior to stimulation (*p < 0.05 by multiple comparisons one-way ANOVA with Dunnett’s *post-hoc* test). Data are mean±SD; *n* = 8 neurons per experimental group and 327–400 boutons.

Multiple presynaptic targets have been identified for volatile anesthetic actions (Miao et al., 1995; Schlame and Hemmings, 1995; Hemmings et al., 2005; Westphalen et al., 2011, 2013), but they do not fully explain anesthetic-induced reduction in excitatory transmission. As BDNF modulates glutamate release (Figure 2), we tested the role of BDNF in the effects of isoflurane by determining isoflurane-induced changes in activity-dependent BDNF release in live hippocampal neurons using pHluorin fused to the C-terminus of BDNF (BDNF-pH). BDNF release can be induced electrically by high-frequency tetanic stimulation, and BDNF-pH is properly processed, asynchronously released (Figures 3A,B and Supplementary Movie 2), and biologically active following secretion (Matsuda et al., 2009). Exocytosis of BDNF was inhibited ∼47% by isoflurane (Figure 3C) suggesting a novel role in contributing to isoflurane-induced effects of synaptic transmission. Taken together, our data show that BDNF contributes to SV exocytosis (Figure 2) and isoflurane inhibits BDNF release (Figure 3). To delineate the consequence of reduced BDNF release by isoflurane on glutamatergic SV exocytosis, we examined isoflurane-induced reduction of SV exocytosis using vGlut1-pH in the presence of TrkB-Fc to sequester extracellular BDNF. Reducing extracellular BDNF attenuated depression of SV exocytosis by isoflurane (Figure 4) showing a contribution of BDNF signaling to modulation of excitatory transmission by isoflurane.

### Neurons Expressing Val66Met and Met66Met Brain-Derived Neurotrophic Factor Show Prolonged Depression of Synaptic Vesicle Exocytosis by Isoflurane Compared to Val66Val Neurons

**FIGURE 5:**
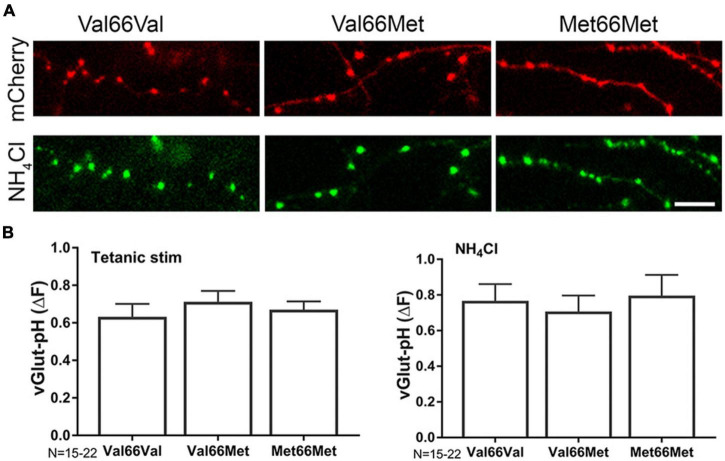
BDNF Val66Met and Met66Met single nucleotide polymorphisms do not affect total synaptic vesicle number or amount of exocytosis. Representative images of 14–16DIV Val66Val (wt), Val66Met, and Met66Met mouse hippocampal neurons transfected with mCherry (**A**-top) and vGlut-pH (with NH_4_Cl alkalization, **A**, bottom). **(B)** Change in fluorescence of vGlut-pH with tetanic stimulation (left) or NH_4_Cl alkalization (right) reflecting amount of exocytosis or total vesicle number showed no significant differences between the three genotypes. Data are mean±SD; *n* = 15–22 neurons per experimental group and 750–1,100 boutons. Scale bar = 5 μm.

Attenuation of excitatory transmission leads to morphological changes in hippocampal dendritic spines (Zhou et al., 2004), and persistent changes in spine structure are associated with cognitive dysfunction in various neurological disorders (Blanpied and Ehlers, 2004). BDNF regulates spine size, and knock-in mice with a BDNF SNP (BDNF Val66Met) have altered spine morphology and density in the medial prefrontal cortex, basolateral amygdala (Yu et al., 2012) and hippocampus (Giza et al., 2018). However, alterations in presynaptic structure and function due to the Val66 genotype have not been investigated. The Val66Met SNP resulted in abnormal intracellular trafficking of BDNF leading to lower regulated secretion of mBDNF (Hariri et al., 2003; Szeszko et al., 2005; Bath and Lee, 2006), but did not directly influence glutamate release or total amount of releasable pool (Figure 5B) as assayed by vGlut1-pH with tetanic stimulation. Presynaptic bouton size and number are difficult to determine in unpaired cells due to heterogeneity of the cell population in culture and architecture of the axon, but qualitative differences were not found between Val66Met and Met66Met boutons compared to Val66Val wild-type boutons (Figure 5A).

**FIGURE 6:**
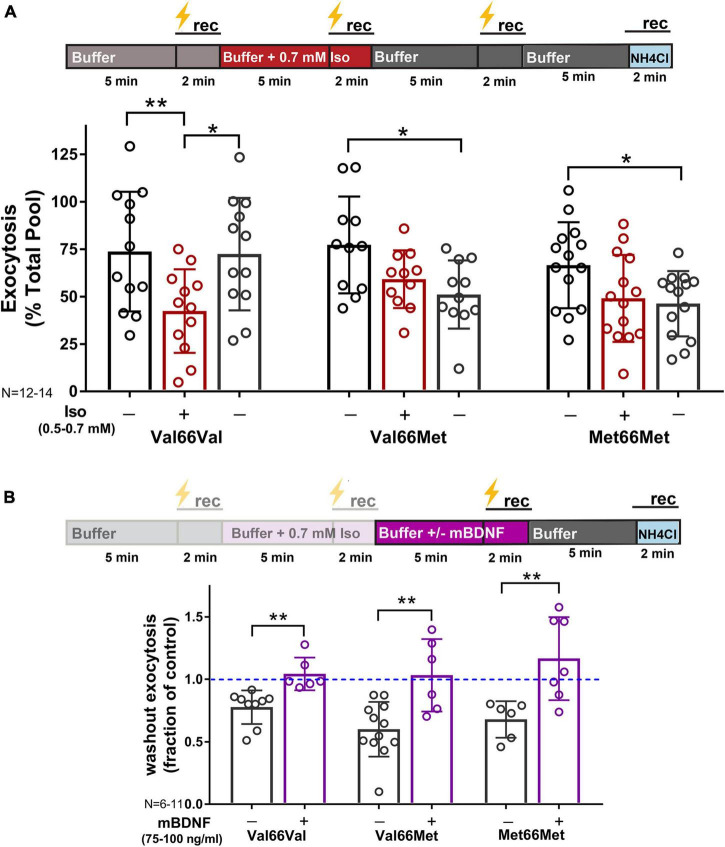
Inhibition of synaptic vesicle exocytosis by isoflurane is acutely irreversible in Val66Met and Met66Met neurons, but rescued with exogenous BDNF. Mouse hippocampal neuron cultures (16DIV) transfected with vGlut-pH and mCherry were perfused with control buffer, 0.5–0.7 mM isoflurane, or mBDNF (75–100 ng/ml) with tetanic stimulation (lightning bolt) as indicated in (**A,B**, top). Tetanic stimulation of synaptic vesicle exocytosis measured with vGlut-pH was inhibited by isoflurane (0.5–0.7 mM) in Val66Val mouse neurons compared to Val66Met and Met66Met neurons (**p < 0.01 by multiple comparisons one-way ANOVA with Dunnett’s *post-hoc* test). Changes in vesicle exocytosis observed in Val66Met and Met66Met neurons after isoflurane exposure did not recover following washout of drug (**A**, bottom) (*p < 0.05 by multiple comparisons one-way ANOVA with Dunnett’s *post-hoc* test). Addition of 75–100 ng/ml of exogenous mBDNF during washout rescued synaptic vesicle exocytosis in Val66Met and Met66Met and increased exocytosis in Val66Val (**B**, bottom) (*p < 0.05; **p < 0.01 neurons by Student’s unpaired *t*-test). Data are mean±SD; *n* = 4–14 neurons per experimental group and 200–700 boutons.

Since BDNF is a target for isoflurane-induced reduction of synaptic transmission (Figure 4), neurons with reduced BDNF levels were less affected by isoflurane exposure (Figure 6A; % inhibition: Val66Val-44%; Val66Met-22%; Met66Met-30%). However, inhibition of SV exocytosis by isoflurane was acutely irreversible in Val66Met and Met66Met neurons compared to Val66Val (Figure 6A) following isoflurane washout at 5 min. These changes were rescued by addition of exogenous mBDNF (Figure 6B), confirming the contribution of the BDNF SNP in producing prolonged structural and functional presynaptic changes with consequences for isoflurane anesthesia.

## Discussion

**FIGURE 7:**
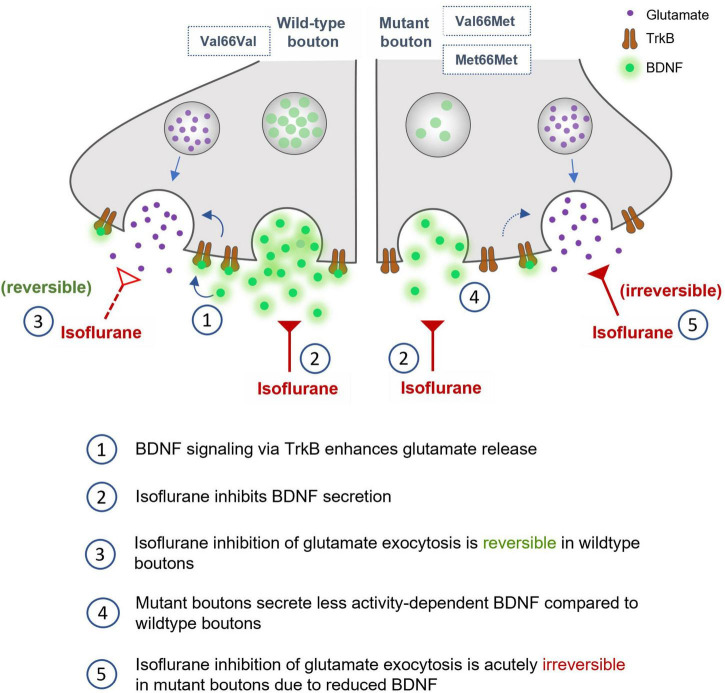
Role of BDNF in isoflurane depression of synaptic transmission in wild-type, Val66Met, and Met66Met hippocampal boutons.

We found that isoflurane inhibits glutamatergic SV exocytosis evoked by high frequency stimulation in part by reducing release of the endogenous neuromodulator BDNF. Neurons with reduced endogenous BDNF levels due to two common BDNF SNPs show lasting reductions in glutamatergic exocytosis following exposure to isoflurane (Figure 7) up to 5 min after washout. Isoflurane effects on BDNF signaling provide a novel mechanism for acute neurophysiological effects that may contribute to lasting impairments in synaptic plasticity and cognitive function based on genotype.

Our findings confirm previous studies (Jovanovic et al., 2000; Tyler and Pozzo-Miller, 2001; Gärtner et al., 2006) that showed that activity-dependent BDNF signaling influences excitatory synaptic transmission by enhancing glutamate exocytosis. We now report that these mechanisms are relevant to isoflurane effects on synaptic function since BDNF release evoked by tetanic stimulation was significantly inhibited by clinically relevant concentrations of isoflurane and contributed to its depression of excitatory SV exocytosis. By removing BDNF as a potential target for isoflurane by preincubation with TrkB-Fc, isoflurane-induced reduction of SV exocytosis was attenuated (Figure 4). These pharmacological effects were further substantiated in neurons with genetically reduced BDNF levels (Val66Met, Met66Met) as they showed reduced sensitivity to isoflurane compared to wild-type Val66Val neurons (Figure 6). Similarly, the acute irreversibility of isoflurane-induced SV exocytosis observed in Val66Met and Met66Met boutons (Figure 6) were recapitulated in Val66Val boutons when BDNF was depleted pharmacologically with TrkB-Fc (data not shown).

Previous studies have implicated proBDNF in anesthetic neurotoxicity in developing neurons and astrocytes (Lu et al., 2006; Head et al., 2009; Pearn et al., 2012; Stary et al., 2015; Liu et al., 2017), but did not investigate the role of mBDNF in established hippocampal cultures. Head and colleagues (Pearn et al., 2012) showed that isoflurane produces neurotoxicity through effects on proBDNF signaling mediated by p75 receptors that promote cell death and attenuate synaptic transmission (Woo et al., 2005; Yang et al., 2009). Upregulating cleavage of proBDNF to mBDNF or direct inhibition of p75 receptors reduces both anesthetic-induced neuronal apoptosis (Pearn et al., 2012) and destabilization of dendritic filopodia in developing neurons (Head et al., 2009), but the direct effects on mBDNF were not reported. Our findings suggests that novel mBDNF mechanisms are involved in the acute and transient effects of isoflurane in reduction of excitatory transmission in established hippocampal boutons.

We found that with high-frequency tetanic stimulation, isoflurane reversibly inhibited excitatory SV exocytosis in wild-type neurons consistent with transient modification of synaptic function. This is consistent with previous studies showing no persistent changes in depolarization-evoked neurotransmitter release for mechanistically distinct anesthetics (Tibbs et al., 1989; Baumgart et al., 2015; Torturo et al., 2019), although distinct mechanisms are posited based on mode of stimulation (Wang et al., 2020). Excitatory transmission is critical to hippocampal synaptic plasticity and can be modulated by anesthetics to produce long-term neurocognitive consequences (Perouansky et al., 2010; Jevtovic-Todorovic et al., 2013). Genetic variation is a risk factor for postoperative neurocognitive decline (Mathew et al., 2007), and the BDNF Val66Met allele is a polymorphism associated with susceptibility to postoperative neurocognitive decline (Mulholland et al., 2014; Silbert et al., 2015; Giattino et al., 2017). This provides a mechanism for changes in synaptic connectivity and function that may persist beyond acute anesthetic actions, possibly contributing to cognitive dysfunction.

The biochemical, anatomical, and behavioral consequences of the human BDNF Val66Met polymorphism *in vivo* are recapitulated in a Val66Met BDNF knock-in mouse model (Chen et al., 2006, 2008). These mice have attenuated long-term potentiation (LTP; Ninan et al., 2010) and reduced hippocampal neuronal soma size and dendritic complexity (Chen et al., 2006). Regulated BDNF secretion is reduced by 18% in Val66Met and 30% in Met66Met neurons (Chen et al., 2006) compared to wild-type, allowing comparisons of “dose-dependent” BDNF effects in disruption of synaptic function by isoflurane. We found that in Val66Met and Met66Met mouse hippocampal boutons isoflurane induced prolonged depression of SV exocytosis compared to Val66Val boutons. Both BDNF and glutamate exocytosis are inhibited by isoflurane anesthesia in wild-type neurons, but a reduced baseline of BDNF levels necessary to reach an estimated threshold may be needed to induce lasting changes in neurotransmitter release. The limits of BDNF levels sufficient to enhance or produce irreversible changes by volatile anesthetics should be further determined by titrating extracellular BDNF levels using TrkB-Fc. Our results suggest that although the contribution of mBDNF to glutamate release is modest (Figure 2), it is a critical component of synaptic function in the presence of isoflurane. Whether these alterations in synaptic function are accompanied by changes in synaptic structure and connectivity will require further study as our study only tested acute reversibility. Consistent with a potential role of regulated BDNF release in synaptic transmission, the Met66Met genotype impairs NMDA receptor-mediated synaptic transmission and plasticity compared to wild-type in the hippocampus (Ninan et al., 2010). Reduced synaptic transmission due to the BDNF SNP is region-dependent, as enhanced glutamatergic transmission was observed in the dorsolateral striatum of BDNF Met66Met mice compared to wild-type, but functionally still resulted in impairments of LTP and LTD (Jing et al., 2017).

Our results did not find genotypic differences between Val66Val, Val66Met, or Met66Met boutons in SV total pool size or vGlut1-pH exocytosis with tetanic stimulation, implicating a role for reduced mBDNF in modulating the persistent presynaptic changes by isoflurane. This BDNF-dependent effect on the irreversibility of isoflurane inhibition of SV exocytosis in Val66Met and Met66Met boutons was verified by rescue with exogenous mBDNF (Figure 6). The magnitude of SV exocytosis with mBDNF was higher than that of control stimulations suggesting that basal transmission was sub-maximally stimulated by endogenous mBDNF. Exogenous mBDNF can induce hippocampal plasticity both *in vivo* (Messaoudi et al., 2002) and *in vitro*, with short-term synaptic enhancement occurring at 20 ng/ml *in vitro* (Miao et al., 2021). However, we did not observe an effect below 75 ng/ml most likely due to losses during perfusion affecting peptide delivery. The intersection of BDNF SNPs with persistent isoflurane-induced changes in glutamatergic transmission and plasticity should be confirmed with electrophysiological measurements.

BDNF is a growth factor packaged in and released from large dense-core vesicles. It is constitutively released but can also be released in mature neurons by prolonged depolarization, high-frequency, theta-burst, or tetanic stimulations (Edelmann et al., 2014; Brigadski and Leßmann, 2020) which have physiological correlates in synaptic transmission and plasticity (Hartmann et al., 2001; Matsuda et al., 2009). We studied anesthetic effects on BDNF release in real-time using the fluorescent fusion protein BDNF-pH, which can be released by tetanic stimulation (Kolarow et al., 2007; Matsuda et al., 2009). The fluorescence of BDNF-pH is quenched by the acidic interior of dense core vesicles where BDNF is stored for release and is rapidly unquenched when these vesicles fuse with the plasma membrane. BDNF can be released from axons or dendrites (Kojima et al., 2001; Matsuda et al., 2009) and has diverse presynaptic and postsynaptic modulatory effects on mature glutamatergic synapses consistent with our evidence as a presynaptic target for anesthetics. Using BDNF-pH, Matsuda et al. examined stimulation-evoked BDNF secretion from dissociated rat hippocampal neurons. With brief stimuli, BDNF secretion occurred predominantly at dendrites compared to axons, while prolonged high-frequency stimulation was necessary for vesicular fusion from axons (Matsuda et al., 2009). Based on our stimulation paradigm, these findings support anesthetic-mediated effects on axonal BDNF, but further studies are needed to delineate possible site-specific actions. To explore all possible sites of BDNF release, we did not include blockers of postsynaptic NMDA or AMPA receptors in our protocols as BDNF release from dendritic spines is largely NMDA-dependent (Harward et al., 2016). However, we performed time control experiments with three successive stimulations to exclude postsynaptic potentiation and to ensure fluorophore stability (Supplementary Figure 1).

The mechanisms for acute depression of excitatory synaptic transmission by general anesthetics include reduction of neuronal excitability (Pittson et al., 2004), action potential conduction (Berg-Johnsen and Langmoen, 1986; Mikulec et al., 1998; Wu et al., 2004; Ouyang and Hemmings, 2005), presynaptic Ca^2+^ influx (Baumgart et al., 2015; Koyanagi et al., 2019), synaptic vesicle (SV) exocytosis (Westphalen and Hemmings, 2003, Westphalen and Hemmings, 2006; Torturo et al., 2019), and/or blockade of postsynaptic glutamate receptors (Dickinson et al., 2007). Based on our current findings, further studies should examine the contribution of BDNF and the role of genotype to these mechanisms. We have identified a role for reduced BDNF signaling resulting from the Val66Met SNP, which alters synaptic function following exposure to isoflurane. Whether these changes are permanent or cause lasting harm requires further study. Dissociated hippocampal neurons replicate most of the fundamental cellular and molecular features of synaptic structure and function so our findings are of potential fundamental importance *in vivo*. However, the irreversible deficit in glutamatergic synaptic function produced by isoflurane in the BDNF Met SNPs should be further verified in functional and behavioral assays *in vivo*.

Previous studies have observed genetic variation in vulnerability to postoperative neurocognitive dysfunction. Based on our findings additional studies are needed to confirm whether the effects observed in rodent hippocampal neurons can be extrapolated to mechanistically distinct anesthetics, other brain regions, and other neuronal subtypes, and whether they translate to the clinical setting. These genotype-specific anesthetic effects have potential implications for precision or personalized anesthetic approaches to reduce anesthetic side effects.

## Data Availability Statement

The original contributions presented in this study are included in the article/Supplementary Material, further inquiries can be directed to the corresponding author/s.

## Ethics Statement

The animal study was reviewed and approved by the Institutional Animal Care and Use Committee (IACUC) of Weill Cornell Medicine.

## Author Contributions

JP, HH, and FL conceived and designed the experiments, contributed reagents, materials, and analysis tools, and contributed to the writing of the manuscript. KJ and RW performed the experiments and analyzed the data. All authors contributed to the article and approved the submitted version.

## Conflict of Interest

The authors declare that the research was conducted in the absence of any commercial or financial relationships that could be construed as a potential conflict of interest.

## Publisher’s Note

All claims expressed in this article are solely those of the authors and do not necessarily represent those of their affiliated organizations, or those of the publisher, the editors and the reviewers. Any product that may be evaluated in this article, or claim that may be made by its manufacturer, is not guaranteed or endorsed by the publisher.

## Footnotes

1
http://rsb.info.nih.gov/ij/plugins/time-series.html
