# Supplementary material for: A Common Human Brain-Derived Neurotrophic Factor Polymorphism Leads to Prolonged Depression of Excitatory Synaptic Transmission by Isoflurane in Hippocampal Cultures
Source: Front Mol Neurosci. 2022 Jun 23;15:927149. doi: 10.3389/fnmol.2022.927149 (PMC9260310; doi:10.3389/fnmol.2022.927149)
Supplement: Supplementary file 3 [file Data_Sheet_1.PDF]

Supplementary Material

1.1 Supplementary Figures

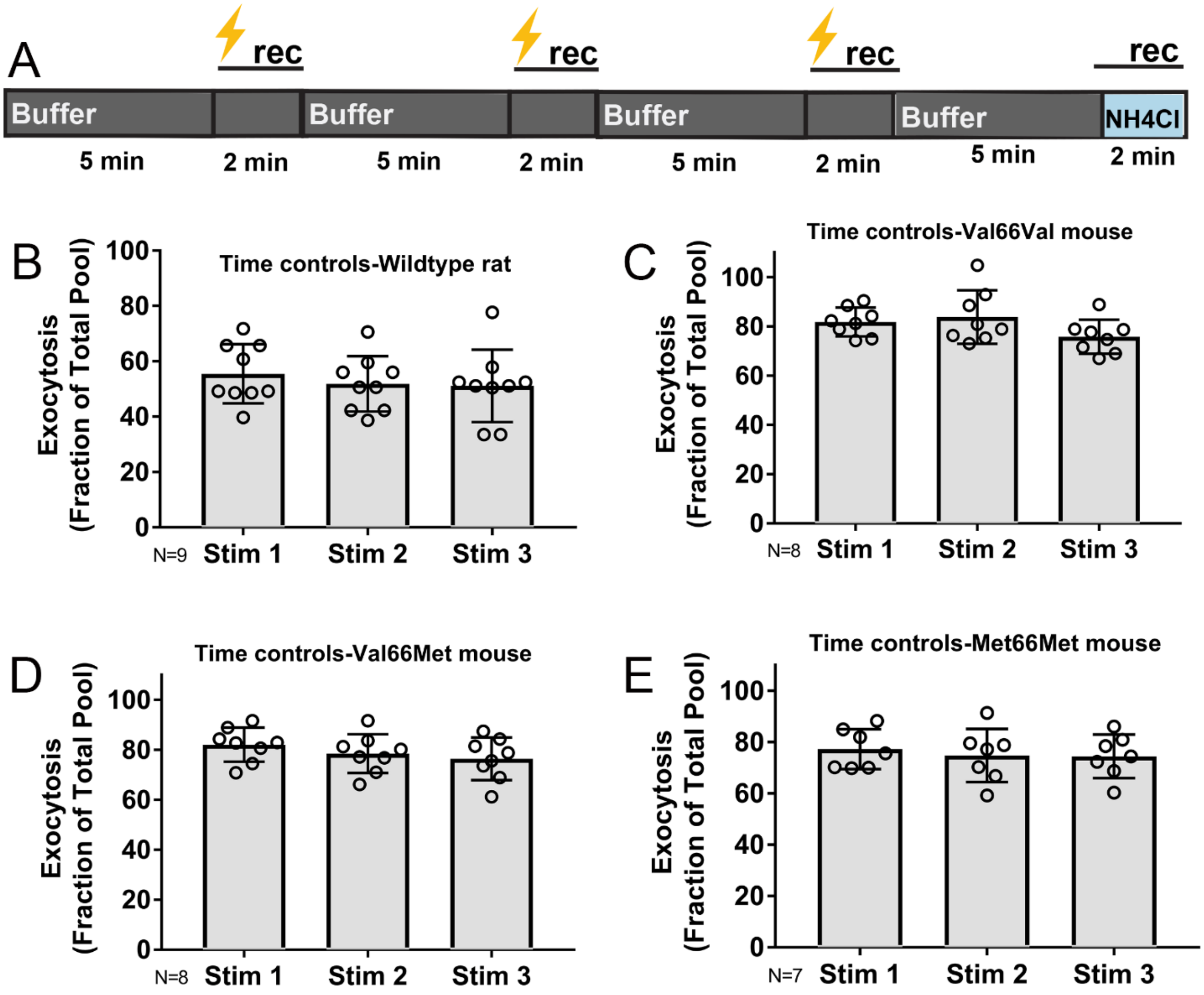

Supplementary Figure 1. Multiple tetanic stimulations do not affect synaptic vesicle exocytosis. Rat or mouse hippocampal neurons (16-18 DIV) are transfected with vGlut-pH and mCherry. Change in fluorescence of vGlut-pH reflecting amount of exocytosis do not show fluorescence decay over time with three subsequent tetanic stimulations **(A)** in wildtype (rat, **B**; mouse, **C**) or mutant mouse (Val66Met, Met66Met; **D**, **E**) cultures.

Tetanic stimulation releases glutamatergic synaptic vesicles. Rat hippocampal neuron cultures (16DIV) transfected with vGlut1-pH and mCherry (not shown) were perfused with Tyrode's control buffer with tetanic stimulation (16 bursts of 50 action potentials at 50 Hz every 2.5 sec). Time-lapse shows changes in bouton fluorescence under tetanic stimulation from which peak fluorescence is used for quantification.

Movie S2. Tetanic stimulation releases BDNF. Rat hippocampal neuron cultures (18DIV) transfected with BDNF-pH and mCherry (not shown) were perfused with Tyrode's control buffer with tetanic stimulation (16 bursts of 50 action potentials at 50 Hz every 2.5 sec). Time-lapse shows release of BDNF (identified as changes in fluorescence) under tetanic stimulation quantified as events.
